# The design of early warning software systems for financial crises in high-tech businesses using fusion models in the context of sustainable economic growth

**DOI:** 10.7717/peerj-cs.1326

**Published:** 2023-04-21

**Authors:** Houfang Guo

**Affiliations:** Suzhou Industrial Park Institute of Services Outsourcing, Jiangsu, Suzhou, China

**Keywords:** High-tech enterprises, Logistic regression model, XGboost, BP neural network, Fusion model

## Abstract

Enterprises are urged to continue implementing the sustainable development strategy in their business operations as “carbon neutrality” and “carbon peak” gradually become the current stage’s worldwide targets. High-tech businesses (HTE) need to be better equipped to manage financial risks and avoid financial crises in the face of severe market competition. The most popular machine learning models—logistic regression, XGBoost, and BP neural networks—are chosen as the base models in this study. The three models are combined using the stacking method to train and forecast the fusion models while offering other researchers some basic model research ideas. The financial crisis early warning (FCEW) of HTE is built concurrently by contrasting the fusion of various quantitative basis models and the fusion procedures of voting and averaging. The outcomes demonstrate that the fusion model outperforms the single model in terms of performance, and the stacked fusion model has the best early warning impact. By comparing and comparing the effect of three fusion models on financial crisis warnings of high-tech enterprises, it makes up for the defect of low accuracy of traditional forecasting methods. It improves the sustainable development path of enterprises.

## Introduction

In the process of various financial activities of enterprises, due to various difficult or unpredictable and uncontrollable factors, the actual financial results of enterprises deviate from the expected financial results, so there is the opportunity or possibility of economic losses. International economic activities tend to move from the movement of commodities to the movement of production factors and achieve the same effect as international trade. The latter emphasizes the incompleteness of the market and deduces the formation of foreign direct investment. China’s economic development has entered a new normal stage; economic growth has entered the medium-low speed stage. Under the background of continuous development and optimization of economic structure, various industries face fierce competition, and sustainable development has gradually become the goal of each enterprise (Batkovsky et al., 2016). It is necessary to clarify the policy direction of sustainable development of enterprises, pay more attention to the coordinated development of economic growth and society, improve the quality of economic growth, and promote the comprehensive development of enterprises. High-tech enterprises (HTE) play an important role in promoting upgrading industrial structure and improving the country’s core competitiveness. HTE has the advantages of fast iteration, rapid development and high income, which also have increased risk characteristics. For HTE, a specific high-risk industry, how to build a scientific and effective FCEW model is of great significance to the HTE themselves, market supervision departments, investors and creditors.

The research on financial crisis prediction mainly includes constructing a prediction model and setting the prediction index. There are two prediction methods: one is based on statistical theory, and the other is a machine learning method represented by artificial intelligence. Generally, financial indicators are used to predict the financial crisis, and the indicators to measure the financial crisis’s value are the property rights ratio and net interest rate of equity. Xu, Qi & Sun (2020) employed logistic regression analysis to forecast businesses’ financial status. The model based on statistics has greater sample requirements, and when the number of samples is high, the generalization ability and prediction effect may be subpar. Chen et al. (2022) built the L1/2 regularised logistic regression model, which prevented the flaw of multicollinearity interference between financial indicators and enhanced the model’s generalizability and FCEW accuracy. Lapedes & Farber (1987) adopted the neural network method for early warning of the bank credit crisis. Sun & Lei (2021) constructed BP-FCEW, which can effectively measure the quality and operating performance of listed enterprises and give financial crisis warning to listed enterprises. Wang et al. (2020) built the twin-SVM model based on the unbalanced sample characteristics of various financial conditions to study the FCEW of Chinese GEM-listed companies. Zhou & Zhang (2019) studied the delisting risk early warning of three *ST companies using the improved equal intercept transform radar map evaluation model. While Aalen model was developed to forecast financial distress after research on the association between default probability and economic early warning signs of Chinese manufacturing listed companies. Zheng (2019) proposed an FCEW model based on rough set theory and least squares SVM for manufacturing listed companies. Some scholars use the Kalman filter algorithm to build a zombie enterprise financial crisis dynamic early warning model or use the group bridge method to select important risk indicators to construct logistic regression early warning model. Yang, Zhou & Sun (2019) proposed using the Benford law to test financial data quality and build a Benford logistic FCEW combining Benford factor variables with financial index variables.

Moreover, some scholars adopted the nonlinear SVM method to early-warning the financial crisis of HTE, which proved that the model has high accuracy in the FCEW of non-listed HTE (Yoon & Park, 2014). However, the selection of financial crisis prediction indicators mainly stays on financial indicators, and non-traditional financial information has not been paid enough attention. Most studies used the fuzzy comprehensive evaluation, logistic regression, and BP neural network (Shang, Lu & Zhou, 2021; Zhang, Xian & Fang, 2019). Still, the learning time of these models is long and the learning accuracy is not high, which affects the prediction effect.

With the continuous improvement of the computer model and intelligent methods, the problem of artificial learning is poor. Many practices have also proved that the effect of the model constructed by the integrated learning method is better than that of the single model (Wang & Wu, 2017). However, there are not many applications of the ensemble learning method in the research of enterprise FCEW. West, Dellana & Qian (2005) found that the enterprise FCEW constructed by bagging and boosting methods performed better than the neural network model. Xie, Li & Han (2005) used Bagging and Boosting algorithms, respectively in the construction of FCEW and pointed out that the performance of the model was better than that of a single model.

From the above analysis, we can see that the model constructed by the ensemble learning method often performs better than a single model. Ensemble learning improves the prediction performance of a single model by training multiple models and combining their predictions. Currently, there is no research on applying the ensemble learning method in predicting the financial condition of high-tech enterprises. Therefore, this article takes the fusion learning method to establish the FCEW model of HTE, hoping to supplement the existing research results. In addition, this article studies and analyzes the causes of the financial crisis in HTE from many aspects, establish an early warning index system and constructs a fusion model as the FCEW model of HTE.

## Design and optimization of different fcew models

To effectively compare the effects of other models, the 50-fold cross-validation method is used to select parameters in model parameter optimization, where SMOTE is used to process unbalanced data, and the training set and test set are divided reserved in advance with a ratio of 7:3.

### Logistic regression model

#### Model description

Logistic regression is a branch of the generalized linear model and an extension of an ordinary linear regression model. The linear regression model establishes a linear combination model to fit the relationship between variables when there is a linear relationship between some variables in the model. In general, the relationship between variables can be shown by the following expression:

(1) $${\rm g}\left( {\rm x} \right) = {{\rm g}_{\rm \alpha }}\left( {\rm x} \right) = {{\rm \alpha }_0} + {{\rm \alpha }_1}{{\rm x}_1} + {{\rm \alpha }_2}{{\rm x}_2} + \cdots + {{\rm \alpha }_{\rm i}}{{\rm x}_{\rm i}}$$

where, ${{\rm x}_{\rm i}}$ represents the ${\rm i}$-th independent variable, and ${{\rm \alpha }_{\rm i}}\left( {{\rm i} = 1,2 \cdots } \right)$ is the estimate of the variable parameter.

In fact, the FCEW of HTE studied in this article is a two-classification evaluation of the financial situation of listed companies to determine whether there is a financial crisis.

The following mathematical relations can be expressed as follows:

(2) $$\ln \left( {\displaystyle{{{{\rm p}_{\rm i}}} \over {1 - {{\rm p}_{\rm i}}}}} \right) = {{\rm b}_0} + \sum\limits_{{\rm K} = 1}^{\rm n} {\mkern 1mu} {{\rm b}_{\rm k}}{{\rm x}_{{\rm ki}}}.$$

Among them, the ${{\rm p}_{\rm i}} = {\rm p}\left( {{{\rm y}_{\rm i}} = 1{\rm \mid }{{\rm x}_{1{\rm i}}},{{\rm x}_{2{\rm i}}}, \cdots ,{{\rm x}_{{\rm ki}}}} \right),{{\rm p}_{\rm i}}$, ${{\rm p}_{\rm i}}$ represents the probability of events with an independent variable of ${{\rm x}_{1{\rm i}}},{{\rm x}_{2{\rm i}}}, \cdots ,{{\rm x}_{{\rm ki}}}$. ${{\rm b}_0}$ is the intercept and ${{\rm b}_{\rm k}}$ is the independent coefficient.

After simplifying Formula (2), we can get the following results:

(3) $${\rm p} = \displaystyle{1 \over {1 + {{\rm e}^{ - {\rm z}}}}}$$

(4) $${\rm z} = {{\rm b}_0} + \sum\limits_{{\rm k} = 1}^{\rm n} {\mkern 1mu} {\mkern 1mu} {{\rm b}_{\rm k}}{{\rm x}_{\rm k}}$$

According to Formula (3), The logistic regression model represents the increasing function of z. Taking the limit of P, the following results can be obtained. Among them, the ${{\rm p}_{\rm i}} = {\rm p}\left( {{{\rm y}_{\rm i}} = 1{\rm \mid }{{\rm x}_{1{\rm i}}},{{\rm x}_{2{\rm i}}}, \cdots ,{{\rm x}_{{\rm ki}}}} \right),{{\rm p}_{\rm i}}$, ${{\rm p}_{\rm i}}$ represents the probability of events with an independent variable of ${{\rm x}_{1{\rm i}}},{{\rm x}_{2{\rm i}}}, \cdots ,{{\rm x}_{{\rm ki}}}$. ${{\rm b}_0}$ is the intercept and ${{\rm b}_{\rm k}}$ is the independent coefficient.

(5) $$\eqalign{& \mathop {\lim }\limits_{{\rm z} \to - \infty } {\mkern 1mu} {\rm p} = \mathop {\lim }\limits_{{\rm z} \to - \infty } \displaystyle{1 \over {1 + {{\rm e}^{ - {\rm z}}}}} = 0 \cr & \mathop {\lim }\limits_{{\rm z} \to + \infty } {\mkern 1mu} {\rm p} = \mathop {\lim }\limits_{{\rm z} \to + \infty } \displaystyle{1 \over {1 + {{\rm e}^{ - {\rm z}}}}} = 1}$$

The better the financial status of the companies, the closer the *p*-value in the model is to 0. In this case, the situation of companies falling into a financial crisis is deficient; On the contrary, for the company with a worse financial situation, the *p*-value in the model is closer to 1, which indicates that the company may have been in a financial crisis.

#### Regularization parameter learning

**Figure 1:**
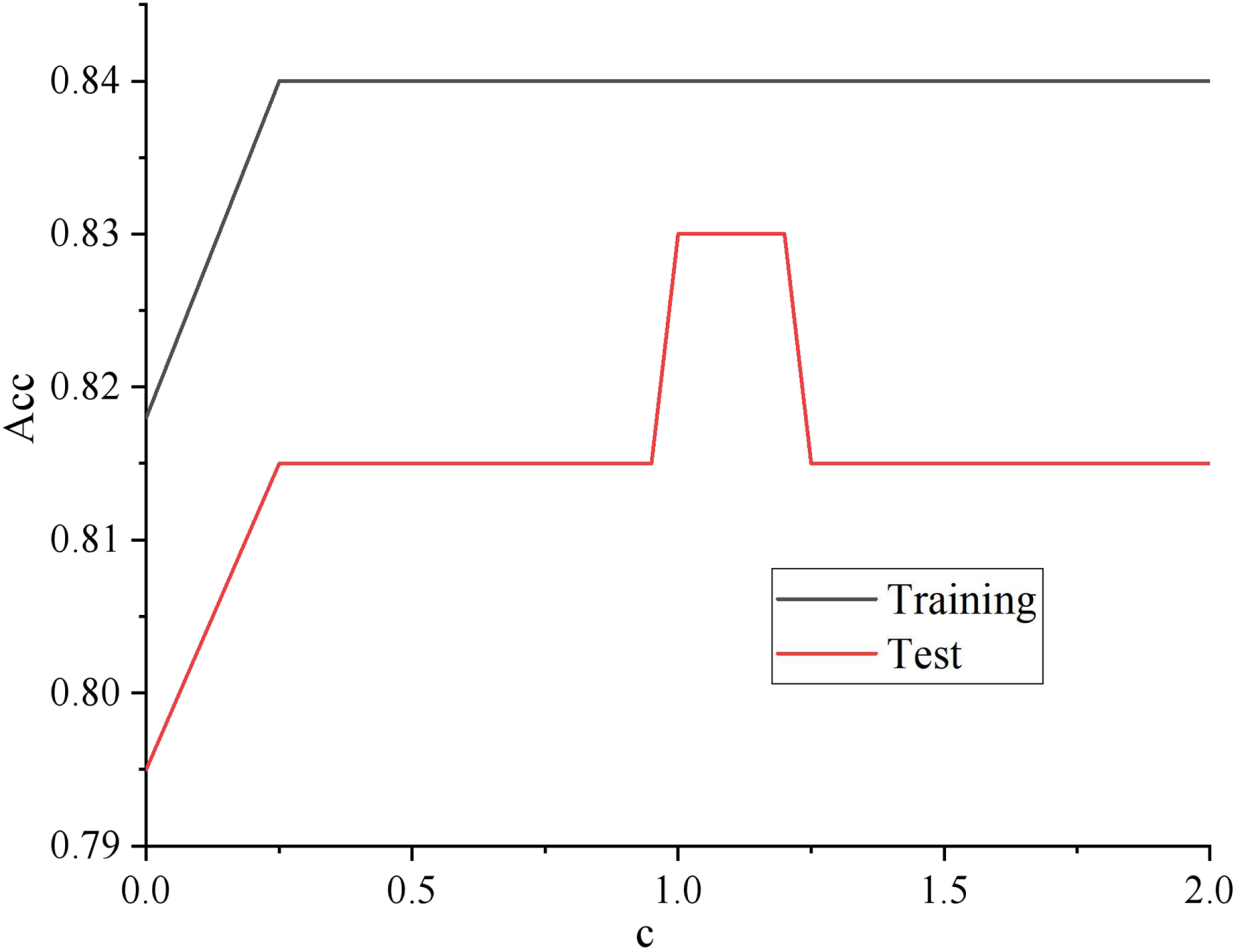
Regularized parameter learning curve.

The learning curve adjusts the regularization parameters. The results are shown in Fig. 1.

As shown in Fig. 1, with the increasing of regularization parameter c, the intensity of regularization becomes smaller, whose performance is getting better and better in both the training set and test set. Until about 0.12, the performance on the training set still showed an upward trend. Too big a step can cause a jolt in the learning curve, but the performance on the unknown data set began to decline. The model has the problem of over-fitting, so it can be assumed that c is better set to 1.2.

### XGboost model

#### Model description

XGboost algorithm can be classified as boosting algorithm in essence. As a lifting tree model, it integrates many tree models to generate the final robust classifier on the principle of boosting algorithm integrating weak classifiers to generate strong classifiers. Every time a tree is generated, the predicted residuals need to be fitted. To predict the scores of a sample, the corresponding scores need to be added at the leaf nodes of each tree, which is the predicted value of the sample.

The expression of XGboost model is as follows:

(6) $$\widehat {\rm y} = \sum\limits_{{\rm k} = 1}^{\rm K} {\mkern 1mu} {{\rm f}_{\rm k}}\left( {{{\rm x}_{\rm i}}} \right)$$

Among them, ${{\rm f}_{\rm k}}\left( {{{\rm x}_{\rm i}}} \right)$ is one of the regression trees, which is also the ${\rm k}$-th base decision tree.

The loss function is defined as:

(7) $${\rm Obj} = \sum\limits_{{\rm i} = 1}^{\rm n} {\mkern 1mu} {\rm l}\left( {{{\rm y}_{\rm i}},{{\widehat {\rm y}}_{\rm i}}} \right) + \sum\limits_{{\rm k} = 1}^{\rm K} {\mkern 1mu} \Omega \left( {{{\rm f}_{\rm k}}} \right)$$

Formula (7) has two terms. It is easy to see that the smaller the difference is, the better the model performs. The overfitting problem of the model is often caused by only optimizing this item; The structural risk loss function is used to measure the complexity of the model, which is also a regularization term. Generally speaking, the larger the value, the better the performance, but the tremendous value may also cause the over-fitting problem of the model. Therefore, balancing the two terms of Formula (7) is necessary to achieve better model performance and avoid over-fitting problems.

As mentioned above, the newly generated tree (*i.e*., the freshly learned function) in the XGboost algorithm should fit the predicted residual. Then, the expected score can be expressed as:

(8) $$\widehat {\rm y} _{\rm i}^{( {\rm t} ) } = \widehat {\rm y} _{\rm i}^{( {{\rm t} - 1} )} + {{\rm f}_{\rm t}}\left( {{{\rm x}_{\rm i}}} \right)$$

Therefore, the objective function of the ${\rm t}$-th iteration is:

(9) $${\rm Ob}{{\rm j}^{\rm t}} = \sum\limits_{{\rm i} = 1}^{\rm n} {\mkern 1mu} {\rm l}\left( {{{\rm y}_{\rm i}},\widehat {\rm y}_{\rm i}^{({\rm t} - 1)} + {{\rm f}_{\rm t}}\left( {{{\rm x}_{\rm i}}} \right)} \right) + \Omega \left( {{{\rm f}_{\rm t}}} \right) + \; {\rm constant}$$

#### Parameter optimization

**Figure 2:**
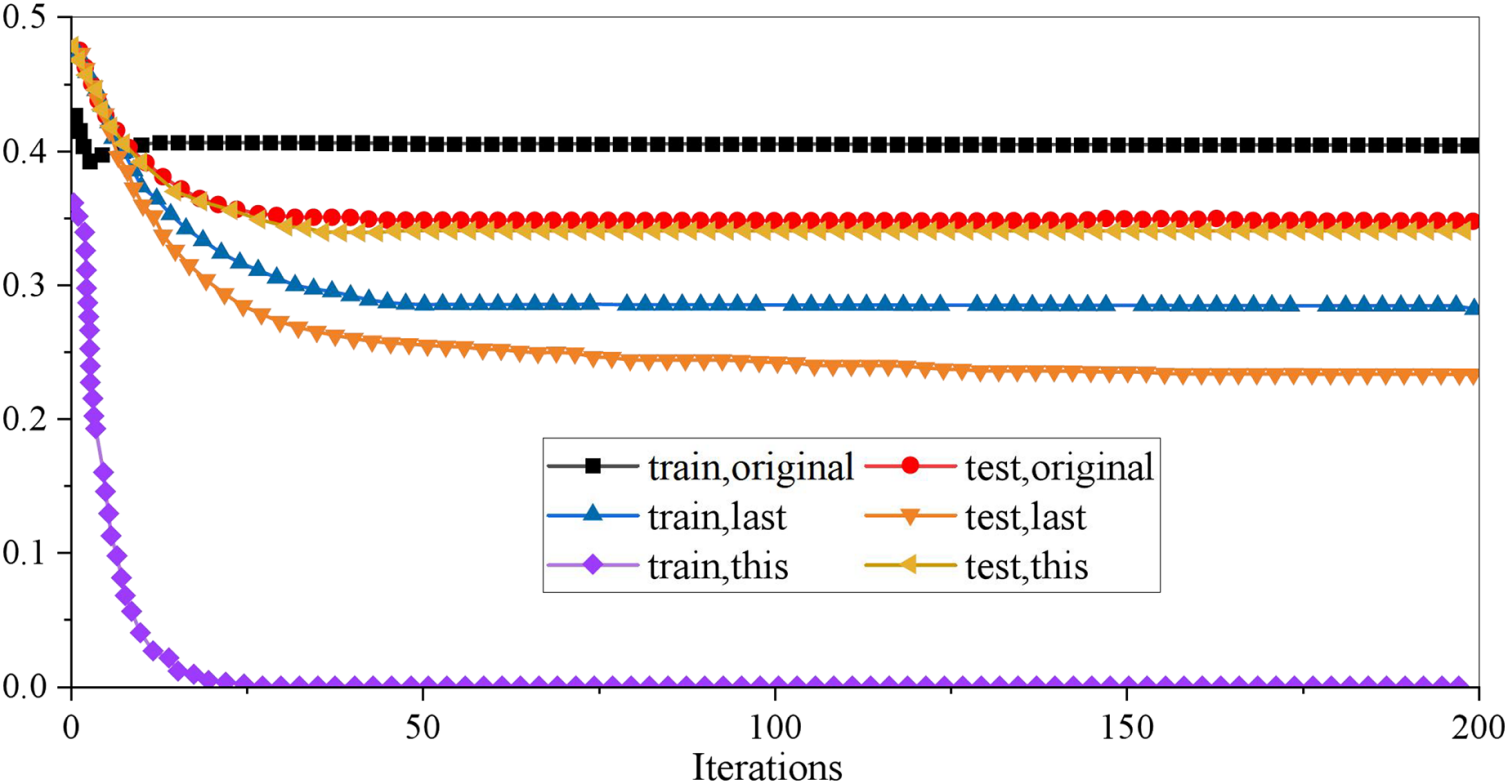
Result of XGboost parameter adjustment.

Optimizing the model’s parameters is necessary to improve the generalization ability of the XGboost model on unknown data sets. In the XGboost model, there are three kinds of super parameters: general parameters, booster parameters and learning objective parameters, which are used to control the fitting data ability of the model. Booster parameters are mainly used to control the iterative process of each base decision tree, including maximum depth, learning rate, and minimum cotyledon sample weight. The interaction of many parameters may reduce the training time and accuracy of the model. In careful consideration of the data set used in this article, eight of the parameters adjusted in this empirical study are involved. In the actual training, we will first make a baseline demo to explore the model’s potential as quickly as possible so we can focus on integrating features and the model in the later period. Therefore, only eight parameters are set. Learning objective parameters control the ideal optimization objectives in each iteration process. The research in this article belongs to a two categories problem and needs to predict the probability of a financial crisis of HTE in unknown data sets. Therefore, the objective (minimum loss function) parameter is binary: logistic. The results are shown in Fig. 2.

It can be seen from the curve that the original XGboost model is in the state of overfitting, so it is decided to optimize it to make the results of the training set and the test set as close as possible. Suppose the effects on the test set cannot be increased. In that case, it is also an excellent choice to reduce the results on the training set, that is, to make the model not completely dependent on the training data and increase the model’s generalization ability. To describe the pruning process more clearly, this article uses three groups of curves to compare: one group is used to show the results of the original model data, one group is used to show the results of the previous parameter adjustment, and the last group is used to show the results of the parameters of the model is adjusted. Table 1 shows the optimal values of the eight parameters of the final model.

**Table 1:** Parameter setting of XGboost model.

Parameter label	Meaning of parameters	Optimal value
eta	Learning efficiency	0.06
max_depth	Maximum depth of decision tree	2
gamma	Regular term parameters	0.3
lambda	Regular term parameters	0.3
alpha	Regular term parameters	0.3
colsample_bynode	Node subsampling rate	0.8
colsample_bylevel	Depth subsampling rate	1
colsample_bytree	The proportion of characteristic number in random sampling	0.8

### BP neural network

**Figure 3:**
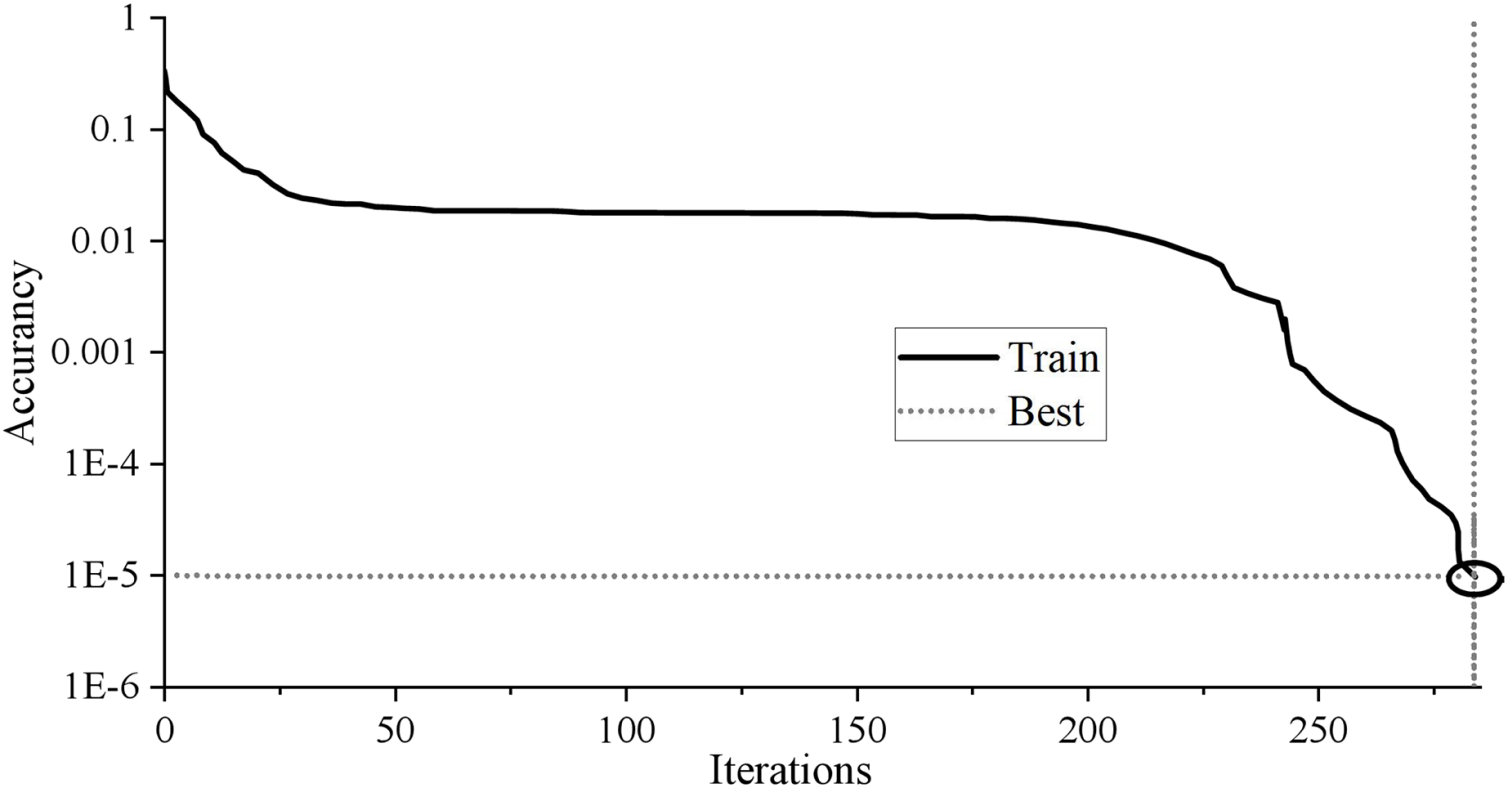
Training results of BP neural network.

It contains seven input nodes and five hidden layer nodes. As long as the network error reaches 0.00001 or the number of iterations reaches 1,000, the network will automatically stop training output. A 7 × 5 × 1 BP neural network topology structure is constructed. The training results are shown in Fig. 3.

The BP neural network training results in Fig. 3 show that the neural network’s training stops after 284 training times. With the continuous decline of MSE, the training error decreases to 9.9205 * 10^−6^ after 284 training times, less than the target error of 0.00001, and the training meets the requirements. The fitting coefficient of sample R is 0.99998, which shows that the fitting effect is perfect. Generally speaking, if the R-value is above 0.9, the fitting effect of BP network training can achieve the expected goal.

## Fusion model

### Index selection

This article takes the enterprises identified as HTE in China’s A-share market as the research object. It takes “Special Processing (ST)” as the standard to divide normal financial and financial crisis enterprises. A total of 462 HTE from 2018 to 2020 are selected as empirical samples, of which 42 are ST enterprises and 420 are nonST enterprises. The ratio of the two kinds of samples is 1:10, which has a serious imbalance. The experiment is divided into a training set and a test set by a five-fold cross-validation method, with “−1” for normal financial enterprises and “+1” for financial crisis enterprises.

Combined with the characteristics of high and new technology enterprises, such as significant intangible assets and strong research and development ability, the financial crisis of high and new technology enterprises is judged from many aspects. By referring to the existing research (Du, 2021), this article initially selects 30 financial indicators of the enterprise as the alternative indicators. Before the experiment, 13 indicators with missing data were eliminated, and 17 were retained as the model’s input variables. The sample indicators are shown in Table 2.

**Table 2:** Sample indicators.

Variable	Name	Variable	Name
X1	Current ratio	X10	Turnover rate of current assets
X2	Quick ratio	X11	Turnover rate of fixed assets
X3	Net cash flow/current liabilities	X12	Turnover rate of total assets
X4	Asset liability ratio	X13	Net cash content of operating income
X5	Equity multiplier	X14	Return on assets
X6	Equity ratio	X15	Net profit margin of total assets
X7	Capital accumulation rate	X16	Return on net assets
X8	Growth rate of total assets	X17	Profit before interest and tax
X9	Turnover rate of accounts receivable		

The data in this article are from the Guotai’an financial database, and the financial index data of the sample enterprises in the 2 years before the financial crisis (T-2) are selected as the experimental input. Before the empirical analysis, the data is normalized to obtain new financial index data ${\rm x}_{\rm i}^{\rm *},{\rm i} = 1,2, \cdots ,17$.

### Evaluation method

The model’s prediction accuracy is a mainstream evaluation index in the existing research. If the accuracy rate is higher, the model has better warning performance. Predicted results usually contain the correct sample $\left| {\rm T} \right|$ and error sample $\left| {\rm F} \right|$. The prediction of Accuracy A is calculated as follows:

${\rm Accurancy} = \displaystyle{{\left| {\rm T} \right|} \over {\left| {\rm T} \right| + \left| {\rm F} \right|}}$

With a class of non-equilibrium samples, it is not scientific and effective to use the prediction accuracy as the evaluation index of the model because the prediction results tend to incline to the majority of samples, and it is easy to ignore a small number of samples, leading to uneven prediction results. Therefore, this article also introduces the geometric mean accuracy rate G (g-mean) and F (F1_score) as evaluation indexes to evaluate the performance of the FCEW of HTE. The construction process of the three evaluation indicators is as follows:

Assuming that $\left| {{\rm F}{{\rm S}_{{\rm min\; }}}} \right|$ and $\left| {{\rm F}{{\rm S}_{{\rm maj\; }}}} \right|$ made of ST companies sample differentiating respectively divided into ST sample enterprise and the ST enterprises made samples of differentiating into non-ST the number of samples. $\left| {{\rm T}{{\rm S}_{{\rm min}}}} \right|$ and $\left| {{\rm T}{{\rm S}_{{\rm maj}}}} \right|$ of the ST and the ST enterprises, the number of samples correctly, usually expressed using a confusion matrix, as shown in Table 3.

**Table 3:** Confusion matrix of binary data sets.

	The model is classified as a sample of enterprises without a financial crisis	The model is classified as a sample of enterprises with the financial crisis
It is actually a sample of enterprises without a financial crisis	$\left| {F{S_{{\rm maj\; }}}} \right|$	$\left| {F{S_{{\rm min\; }}}} \right|$
It is actually a sample of enterprises with financial crisis	$\left| {T{S_{maj}}} \right|$	$\left| {T{S_{{\rm min}}}} \right|$

Using the matrix, we can divide the four situations in the experiment. Then we can get the Response rate RE, the Specificity S and the Precision P through the relevant calculation:

(10) $${\rm RE} = \displaystyle{{\left| {{\rm T}{{\rm S}_{{\rm min}}}} \right|} \over {\left| {{\rm T}{{\rm S}_{{\rm min}}}} \right| + \left| {{\rm F}{{\rm S}_{{\rm maj}}}} \right|}}$$

(11) $${\rm SP} = \displaystyle{{\left| {{\rm T}{{\rm S}_{{\rm maj}}}} \right|} \over {\left| {{\rm T}{{\rm S}_{{\rm maj}}}} \right| + \left| {{\rm F}{{\rm S}_{{\rm min}}}} \right|}}{\rm \; }$$

(12) $${\rm P} = \displaystyle{{\left| {{\rm T}{{\rm S}_{{\rm min}}}} \right|} \over {\left| {{\rm T}{{\rm S}_{{\rm min}}}} \right| + \left| {{\rm F}{{\rm S}_{{\rm min}}}} \right|}}{\rm \; }$$

Then, ${\rm G}$ and ${\rm F}$ can be calculated as follows:

(13) $${\rm G} = \sqrt {{\rm RE} \times {\rm SP}}$$

(14) $${\rm F} = \displaystyle{{2 \times {\rm RE} \times {\rm P}} \over {{\rm RE} + {\rm P}}}$$

Among them, G comprehensively investigates the model’s prediction performance for two kinds of samples with high reference values when the data is unbalanced. If G is large, the model’s accuracy is high, and *vice versa*. However, F mainly investigates the model’s prediction performance for several samples. If F is more significant, it indicates that the model performs better in predicting financial crisis samples and *vice versa*.

The area under the ROC curve (AUC) is introduced to quantitatively evaluate the classifier’s performance. AUC measures the probability of placing positive samples ahead of negative examples, regardless of the domain value. The larger the area of AUC, the better the classification effect.

### Results and discussion

The boosting algorithm, where the learner is associated with Boosting, the latter one needs to learn from the previous one. Stacking is the result of a greater generalization in integrated learning. Stacking results from the aggregation method’s generalization in ensemble learning, which belongs to a hierarchical framework. Suppose a two-layer stacking model input the initial training set in the first stage, let multiple base learners learn and fit, and takes the output results as the input of the second stage classifier (meta leamer) for retraining. The final prediction result is obtained. The feature of this model is that it uses the prediction of the first stage as the feature of the prediction of the next level. In this article, two common model fusion methods, voting and averaging, are used to compare, respectively. The stacking method is an automated, large-scale integration policy. Overfitting can be effectively combated by adding regularization terms, and it does not require too much parameter tuning and feature selection. It is helpful for comparison and experimentation in this study.

Because the XGboost model did well in the previous research and was more stable, the prediction results of the three basic models are used as the characteristic variables that are substituted into the XGboost model to train it.

Any two of the three base models are fused. Among them, the fusion model of logistic regression and XGboost is named “model A,” the fusion model of XGboost and BP is named “model B,” and the fusion model of logistic regression and BP’s neural network model is called “model C”. The stacking fusion, voting, and averaging method fuse three single models: fusion model D.

The final three single models are compared and analyzed, and the results of each model on the test set are shown in Table 4.

**Table 4:** Comparison of model effects.

Model	A	G	F	AUC
Logistic regression model	83.13%	71.40%	66.71%	0.79
XGboost model	83.10%	85.72%	70.63%	0.84
BP neural network model	74.62%	78.60%	59.56%	0.76
Voting fusion model A	84.75%	78.65%	71.04%	0.82
Averaging fusion model A	83.10%	78.60%	68.86%	0.81
Stacking fusion model A	84.7%	85.74%	72.73%	0.85
Voting fusion model B	72.93%	71.46%	55.62%	0.72
Averaging fusion model B	81.42%	78.60%	66.72%	0.80
Stacking fusion model B	84.71%	85.76%	72.70%	0.85
Voting fusion model C	76.37%	64.38%	56.32%	0.72
Averaging fusion model C	78.07%	64.33%	58.10%	0.73
Stacking fusion model C	81.40%	78.62%	66.71%	0.80
Voting fusion model D	84.76%	85.75%	72.70%	0.85
Averaging fusion model D	84.72%	78.62%	71.00%	0.83
Stacking fusion model D	86.42%	92.90%	76.58%	0.89

Compared with the three single models, the accuracy of XGboost and logistic regression is about 83%, which is better than BP and even the Optimized BP neural network model. It can be seen that the recall rate of XGboost reaches 80%, which is equal to the optimized BP neural network model, and better than logistic regression model and BP neural network model. Next, the accuracy rate is still better with XGboost and logistic regression, in addition. Compared with each model’s AUC value, the XGboost model’s AUC value is 0.84, which is slightly better than the others. Even the optimized BP neural network model is only about 0.80.

Compared with the fused model, when the two single models are fused, the performance of models A, B, and C of the stacking fusion method is better than that of the other two fusion methods, which is more robust while maintaining a higher recall rate. The fusion effect of the voting fusion method is better when two single models are fused. In addition, through the comparison, we can see that the performance of stacking fusion method fusion model B is better than the other two models using the stacking fusion method in all aspects, which shows that the performance of a single model is better. When the performance of a single fusion model is closer, the performance of stacking fusion is better.

In addition, the accuracy of the stacking fusion model is 86.42%, which is slightly better than the voting fusion model and averaging fusion model; The accuracy of the three is close, but in the F that we are more concerned about, the stacking fusion model reaches 92.9%, that is to say, in the financial crisis enterprises given by the test set, more enterprises can be predicted more accurately, which is conducive to our research; At the same time, the AUC value of stacking fusion model is 0.887, which is better than other two fusion models.

**Figure 4:**
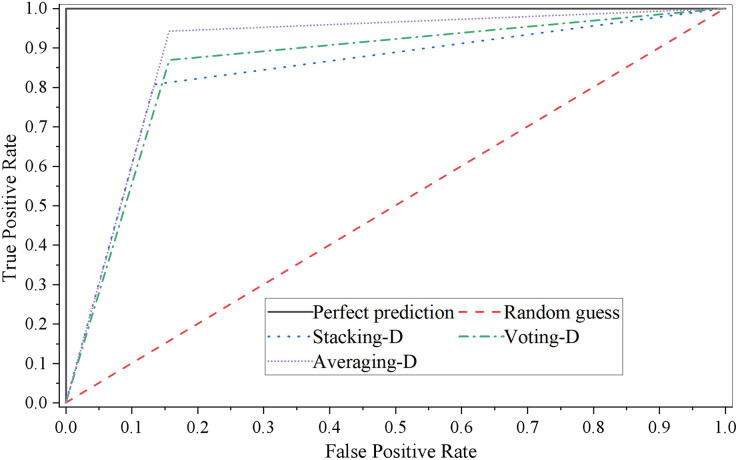
ROC curve of fusion model D.

The ROC curve of three single models is shown in Fig. 4.

It can be seen that the performance of the stacking fusion model on the test set is better, and the accuracy and AUC of the model are relatively higher while ensuring the recall rate. The ROC curve of the stacking fusion model fully covers the ROC curves of the other two fusion models, which indicates that the model constructed by the stacking fusion model has better performance than those built by the two model fusion methods of Voting and Averaging in this article. In addition, we can see that when the threshold is relatively low, the model performance fused by the Averaging model fusion method is similar to that of the stacking fusion model. However, as the threshold increases, the performance of the Averaging model fusion method is gradually worse than that of the other two model fusion methods.

## Conclusion

This study uses the BP neural network model, the XGboost model, and the logistic regression model as the basic models to create several fusion models. The results demonstrate that the two fusion strategies display better results than the averaging model. When compared to the fusion effect of different numbers of base models, it is discovered that the model fusion effect of the stacking approach is better the more disparate the base models are and the closer the performance is. The impact of model fusion also improves as the number of base models rises, but the fusion effect will no longer be optimal at a certain point. It is beneficial for enterprise managers to propose preventive measures for HTE to deal with the financial crisis, ensure the vital interests of other stakeholders, such as investors and creditors, and provide specific guidance for regulators to manage HTE, which has strong theoretical value and practical significance.

## Supplemental Information

10.7717/peerj-cs.1326/supp-1 Supplemental Information 1 Code.

## Additional Information and Declarations

### Data Availability

The following information was supplied regarding data availability: The code is available in the Supplemental File. The data is available at Kaggle and Zenodo: https://www.kaggle.com/code/imranp/starter-synthetic-financial-datasets-cd6449a6-6/data; Open Access. (2023). Synthetic Financial Datasets [Data set]. Zenodo. https://doi.org/10.5281/zenodo.7543591.
